# Morphological and Molecular Identification of the Causal Agent of Anthracnose Disease of Avocado in Kenya

**DOI:** 10.1155/2018/4568520

**Published:** 2018-02-27

**Authors:** S. K. Kimaru, E. Monda, R. C. Cheruiyot, J. Mbaka, A. Alakonya

**Affiliations:** ^1^Department of Plant Sciences, Kenyatta University, P.O. Box 43844, Nairobi, Kenya; ^2^Department of Microbiology, Kenyatta University, P.O. Box 43844, Nairobi, Kenya; ^3^Kenya Agricultural and Livestock Research Organisation, P.O. Box 220, Thika, Kenya; ^4^Jomo Kenyatta University of Agriculture and Technology, P.O. Box 62000, Nairobi, Kenya

## Abstract

Anthracnose disease of avocado contributes to a huge loss of avocado fruits due to postharvest rot in Kenya. The causal agent of this disease has not been clear but presumed to be* Colletotrichum gloeosporioides* as reported in other regions where avocado is grown. The fungus mainly infects fruits causing symptoms such as small blackish spots, “pepper spots,” and black spots with raised margin which coalesce as infection progresses. Due to economic losses associated with the disease and emerging information of other species of fungi as causal agents of the disease, this study was aimed at identifying causal agent(s) of the disease. A total of 80 fungal isolates were collected from diseased avocado fruits in Murang'a County, the main avocado growing region in Kenya. Forty-six isolates were morphologically identified as* Colletotrichum *spp. based on their cultural characteristics, mainly whitish, greyish, and creamish colour and cottony/velvety mycelia on the top side of the culture and greyish cream with concentric zonation on the reverse side. Their spores were straight with rounded end and nonseptate. Thirty-four isolates were identified as* Pestalotiopsis *spp. based on their cultural characteristics: whitish grey mycelium with black fruiting structure on the upper side and greyish black one on the lower side and septate spores with 3-4 septa and 2 or 3 appendages at one end. Further molecular studies using ITS indicated* Colletotrichum gloeosporioides*,* Colletotrichum boninense*, and* Pestalotiopsis microspora* as the causal agents of anthracnose disease in avocado. However, with this being the first report, there is a need to conduct further studies to establish whether there is coinfection or any interaction thereof.

## 1. Introduction

The anthracnose disease is a common disease with wide host range causing severe economic loss. The disease has been reported on a wide variety of crops including avocado, almond, coffee, guava, apple, dragon fruit, cassava, mango, sorghum, and strawberry causing severe economic losses [1–4]. The causal agents of this disease are not clear. However, species of the genus* Colletotrichum* and* Pestalotiopsis* have been reported as causal agents of anthracnose in avocado [5, 6]. Such species includes* C. gloeosporioides*,* C. acutatum*,* C. boninense, C. karstii*,* C. godetiae*[7–11], and* Pestalotiopsis versicolor* [6].

Symptoms of anthracnose appear as pepper spot or speckle spot on immature fruit while still on tree and after fruit harvest during ripening as darkly, black coloured, sunken rounded spots with raised margins on fruit skins [12]. These lesions enlarge rapidly on the fruit skin and into the pulp leading to the death and rotting of the infected plant tissues [13]. The lesions may develop salmon-coloured, sticky spore masses typical of anthracnose diseases of this and many other plant species.

The avocado fruit has a high nutritional value since it contains vitamins (E, B, and C), minerals (potassium, iron, and phosphorus) and a great amount of oil [14]. In Kenya, avocado fruit is one of the most economically important fruits grown by both small and large scale farmers (HCDA, 2016). The fruit is mainly grown for fresh market but there is increasing demand from pharmaceutical, cosmetics, and vegetable oil industries (HCDA, 2016).

Anthracnose caused by* Colletotrichum gloeosporioides *has been associated with severe losses of avocado fruits both in the field and after harvest as compared to* Pestalotiopsis* spp. whose impact is not widely studied [5, 15–18]. Wasilwa et al. [19] reported that over 60% of the Kenyan avocado production cannot be marketed because of damage and low quality of fruits associated with anthracnose disease. Despite the huge losses associated with the anthracnose disease of avocado in Kenya, no cultural and molecular studies have been done to identify the causal agent(s). During the investigation, this paper aimed to identify the causal agent(s) of anthracnose of avocado in Kenya.

## 2. Materials and Methods

### 2.1. Fungal Isolation and Culturing

Samples of infected avocado fruits showing symptoms of anthracnose were collected from study area, Murang'a County, and brought to the laboratory for fungal isolation. The typical symptoms were small blackish spots “pepper spot” to larger blackish spots with raised margin [5]. The samples were cleaned using tap water and blotted to remove excess water. The fruits were surface sterilized using 0.5% sodium hypochlorite for 30 seconds. Small sections of the diseased area were cut aseptically and placed on hardened potato dextrose agar (PDA) in Petri dishes for fungal growth at room temperature (22–25°C). The emerging fungi were subcultured to obtain pure cultures. To obtain pure cultures, single spore isolation was done as follows: cultures were flooded with sterile distilled water and conidia were scraped off the plate using sterilized wire loop and suspended in 1 ml of sterile distilled water. A loopful of conidial suspension was spread evenly on 1.5% (wt/vol) water agar in a Petri dish and incubated at 25°C overnight. Using a sterilized glass needle germinated conidium was transferred onto hardened PDA in 9 cm diameter Petri dish and incubated at 25°C with a 12 h cycle of fluorescent light to induce growth and sporulation. To avoid bacterial contamination 0.5 g/l of streptomycin was added to PDA at molten state of about 50°C [20]. Single spore pure cultures of the pathogen were preserved in the slant universal bottle and stored in the fridge at 4°C for later use.

The fungi,* C. gloeosporioides* and* Pestalotiopsis* spp., were morphologically identified based on cultural and microscopical characteristics using published fungal key [21, 22].

### 2.2. Inoculation, Mycelial Growth, and Sporulation of* C. gloeosporioides* Isolates

Pure cultures of* C. gloeosporioides* preserved in universal bottles were used. Using a sterilized surgical scalpel, sections of mycelial plugs were cut aseptically and placed on hardened PDA on 9 cm diameter Petri dishes and incubated for 10 days for mycelial growth.

Five-millimetre mycelia plugs from the 10-day-old pure isolates of* C. gloeosporioides* were aseptically cut using five-millimetre diameter cork borer and placed individually at the centre of hardened PDA culture in 9 cm diameter Petri dishes. The cultures were incubated at room temperatures of ranges 22–25°C. Mycelia diameters of the isolates were measured at days 2, 4, 6, 8, and 10 after inoculation. On eleventh day, the cultures were flooded with distilled water and scrapped to bring the spores into suspension. The suspension was filtered through double layer cheese cloth to remove mycelia. The spore suspension was serially diluted to 10^−6^ for ease of counting the spores. The spore concentration was determined by use of haemocytometer.

### 2.3. Conidial Morphology and Size

Using a pipette, a drop of spore suspension (10^−6^) was placed on a microscope slide, covered with a cover slip. The spores were stained with lactophenol cotton blue and observed under microscope. The shape of the spores from different isolates was noted and their sizes in terms of length and width were measured using a calibrated ocular slide and stage micrometer.

### 2.4. Determination of Genetic Diversity of Fungal Isolates

#### 2.4.1. DNA Extraction

Pure fungal cultures in potato dextrose agar derived from a single spore from the original isolate were used. An improved fungal extraction protocol as described by Liu et al. [23] was used. About 40 mg of mycelia was placed in an Eppendorf tube containing 2 ml of extraction buffer (Tris-HCl, 100 mM; EDTA, 10 mM; NaCl, 1 M; SDS, 1%; proteinase K, 0.05 mg ml^−1^; pH 8.0) and 10% (v/v) glass beads and ground into powder. The samples were vortexed and incubated at 65°C for 30 minutes. After incubation, the samples were centrifuged at 10,000 ×g for 15 min and supernatant was transferred to a fresh tube. To the supernatant, 150 *μ*l of 3 M guanidine hydrochloride was added and incubated at −20°C for 10 minutes. The samples were centrifuged at 10,000 ×g for 10 minutes. After centrifugation the supernatant was transferred to a fresh tube, and an equal volume of isopropanol was added. Samples were incubated at −20°C for 3 h. The samples were centrifuged for 10 min at 10,000 ×g and thereafter 70% ethanol was added and more centrifugation done for 10 min at 10,000 ×g. The nucleic acid pellet obtained was air dried and dissolved in 50 *μ*l of TE buffer (Tris-HCl, 10 mM, pH 8; EDTA, 1 mM). The nucleic acid dissolved in TE buffer was further treated with 3 *μ*l of RNase (10 mg ml^−1^), to precipitate RNA at 37°C, and the pure DNA obtained was stored at −20°C for later use. The quality of DNA was determined by loading 5 *μ*l of DNA on 1% agarose before running it for 45 volts for 30 minutes and bands were noted by visualization under the UV gel imager.

#### 2.4.2. Polymerase Chain Reactions and Gel Electrophoresis

The DNA extracted from isolates of the pathogens was used as template in polymerase chain reaction. Two sets of primers were used; the first set contained the universal primers ITS1 (5′-TCCGTAGGTGAACCTGCGG-3′) and ITS4 (5′-TCCTCCGCTTATTGATATGC-3′) targeting all fungal isolates while the other set was a primer CgInt (5′-GGGGAAGCCTCTCGCGG-3′) specific to* Colletotrichum gloeosporioides* combined with the universal primer ITS4 (TCCTCCGCTTATTGATATGC) for the identification of the isolates. The amplified regions were subsequently sequenced to obtain sufficient information for the identification of isolates.

#### 2.4.3. Agarose Gel Electrophoresis

A 1.5% agarose gel was prepared using 1x TBE-buffer and stained with 5 *μ*l of SYBR Safe and poured into casting tray having a comb to solidify. The first well of the solid gel was loaded with 1.5 *μ*l 1 Kb marker, followed by 2 *μ*l of each amplified product and a control at the end. The gel was connected to electric voltage of 100 volts for 45 minutes to allow migration of amplified PCR products. The DNA bands formed were visualized under UV light and images photographed using a camera connected to a computer.

#### 2.4.4. DNA Cleaning and Sequencing

The amplified products of the target fragments obtained above were cleaned using the Qiagen PCR cleaning kit according to the manufacturer instructions. The cleaned fragments were submitted for Sanger sequencing at Inqaba Africa Genomic platform in South Africa together with the primers used for amplification (ITS1 and ITS4 and ITS4 and CgInt).

### 2.5. Bioinformatics Analysis

The sequences obtained from Inqaba Africa Genomic platform at South Africa were trimmed before subjecting them to alignment with other Genbank sequences. Gene alignment was done using BioEdit software version while phylogenetic analysis was done by Mega Molecular Evolutionary Genetic Analysis version 7.1.8.

Alignment of sequences of the isolates and phylogenetic analyses was done using Mega 7.18 [24]. The maximum likelihood trees were obtained using the Close-Neighbour-Interchange algorithm while missing data and gaps were eliminated [25]. Clade stability of the resultant phylogenetic tree was based on bootstrap analysis with 1000 replicates [26]. Evolutionary distances in form of number of base substitution per site were computed using the Maximum Composite Likelihood method [24].

## 3. Results

### 3.1. Fungal Isolates

**Figure 1:**
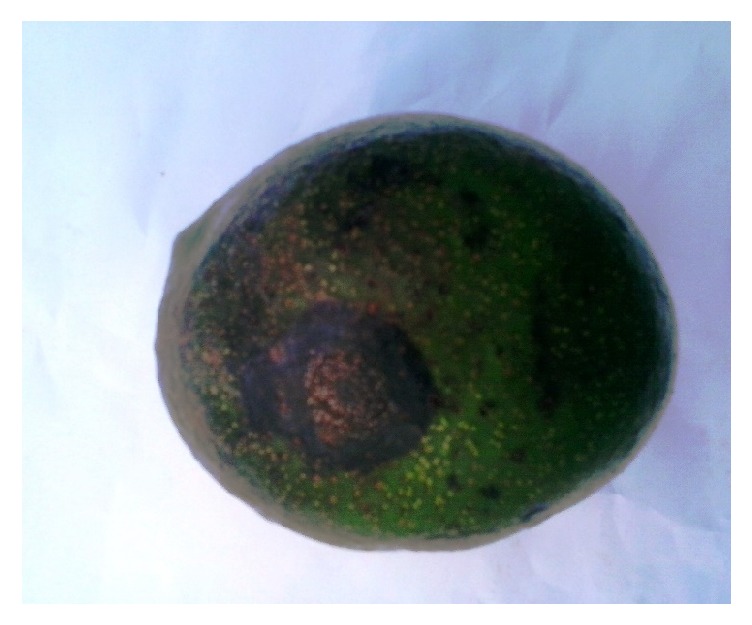
Anthracnose symptoms on avocado fruit.

A total of 80 fungal isolates from diseased avocado fruits showing symptoms of anthracnose (Figure 1) collected from the study area were obtained. The isolates were identified based on cultural morphological characteristic on PDA and spore characteristics as observed under microscope [27, 28].

**Figure 2:**
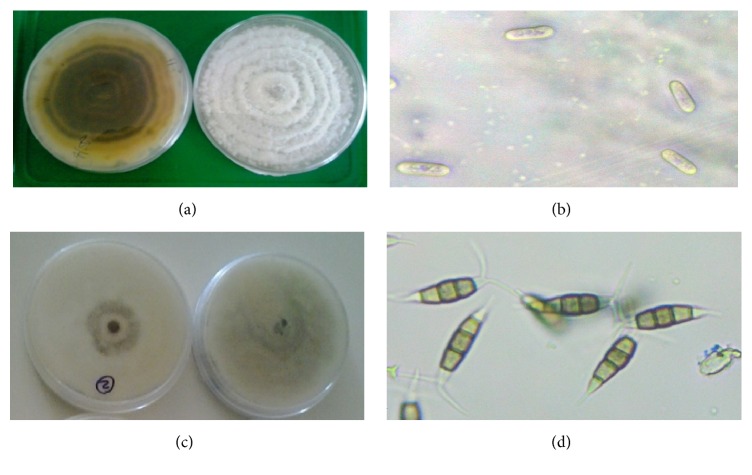
Mycelia of* Colletotrichum gloeosporioides* (a) and* Colletotrichum gloeosporioides spores* (b) (×400) and mycelia of* Pestalotiopsis microspora* (c) and* Pestalotiopsis microspora* spores (d) (×400).

A total of 46 isolates had whitish, greyish, or creamish colour and cottony, velvety mycelium on the top side and greyish cream with circular orange-pinkish colour on the reverse side, Figure 2(a). Their spores were straight with rounded end (Figure 2(b)), typical of* C. gloeosporioides* [29]. The remaining 34 isolates had whitish grey mycelium with black fruiting structure on the upper side and greyish black one on the lower side (Figure 2(c)). Their spores had 3-4 septa and 2 or 3 appendages at one end characteristics of* Pestalotiopsis microspora* [30] (Figure 2(d)).

The isolates were further confirmed through Koch's postulate where ten-day-old pure cultures of the isolates growing on PDA were used to inoculate healthy ripe avocado fruit, Fuerte variety. After two days, a characteristic black spot was formed by both* Colletotrichum* and* Pestalotiopsis *isolates. Each of the reisolated fungi from the diseased fruit showed similar morphological, cultural, and spore characteristics as initial isolates.

The* Colletotrichum* isolates were subjected to more detailed study while the* Pestalotiopsis* spp. was identified further at molecular level using universal primers ITS1 and ITS4.

### 3.2. The Mycelial Growth of* Colletotrichum* Isolates

The* Colletotrichum gloeosporioides* isolates from the study area grew rapidly on the PDA medium covering the whole surface of the Petri dish in 10–12 days after inoculation. The mycelial colour of the isolates varied between whitish grey, whitish cream, and greyish pink on the upper side of the culture (Table 1). Similarly, the lower side of the cultures had creamish grey, greyish orange, and grey (Table 1). In terms of mycelia structure, cottony one was observed in 24 isolates as compared to velvety one observed in 22 isolates (Table 1). There was significant difference (*χ*^2^ = 23.455, df = 2, and *P* < 0.001) among the observed and expected frequencies for the cultural texture and colour of various* Colletotrichum gloeosporioides* isolates (Table 1).

### 3.3. Mycelial Growth of* Colletotrichum gloeosporioides* Isolates

The mycelial diameter of the isolates showed significant differences (*P* < 0.05) throughout the growth period (Table 2). However, they exhibited similar trend in growth with day two having the least and day 10 having the largest diameter per isolate (Table 2). The radial diameter of all the isolates ranged from 0.3 to 0.93 cm and 2.37 to 4.5 cm in day 2 and day 10, respectively (Table 2).

### 3.4. Sporulation of the* Colletotrichum* Isolates

Sporulation of* Colletotrichum gloeosporioides* isolates exhibited a wide range of mean number of spores per isolate ranging from the lowest 0.65 × 10^6^ to the highest 9.00 × 10^6^ spores per ml (Table 2). These mean number of spores per ml differed significantly at *P* < 0.05 among isolates. Isolate s1 had the highest mean number of 9.0 × 10^6^ per ml which was significantly different from the rest at *P* < 0.05 (Table 2). A total of 24 isolates had mean number of spores lower than the tabulated mean of all the isolates of 3.49 × 10^6^ per isolate.

### 3.5. Conidial Morphology and Size

All the spores observed were cylindrical and straight with smooth round end. The spore size varied significantly at *P* < 0.05 among isolates ranging from 3.0 to 5.0 *μ*m in width and 10.3 to 18.2 *μ*m in length (Table 3). The spore widths of isolates s1, s40, and s44 were highest at 5.0 *μ*m and they differed significantly from the rest at *P* < 0.05 (Table 3). Similarly, these isolates produced the longest spores of 18.2, 18.0, and 18.0 *μ*m for isolate s1, s40, and s44, respectively. Isolates s37 and s46, however, produced the smallest spores having a mean of 3.0 *μ*m in width and 10.3 *μ*m in length. Thirty-one isolates produced spores having width within the range of 3.1–3.5 *μ*m which did not differ significantly at *P* ≥ 0.05 (Table 3). Overall, spore size in terms of width and length differed significantly at *P* < 0.05 among isolates.

### 3.6. Phylogenetic Studies of* Colletotrichum gloeosporioides* and* Pestalotiopsis microspora*

**Figure 3:**
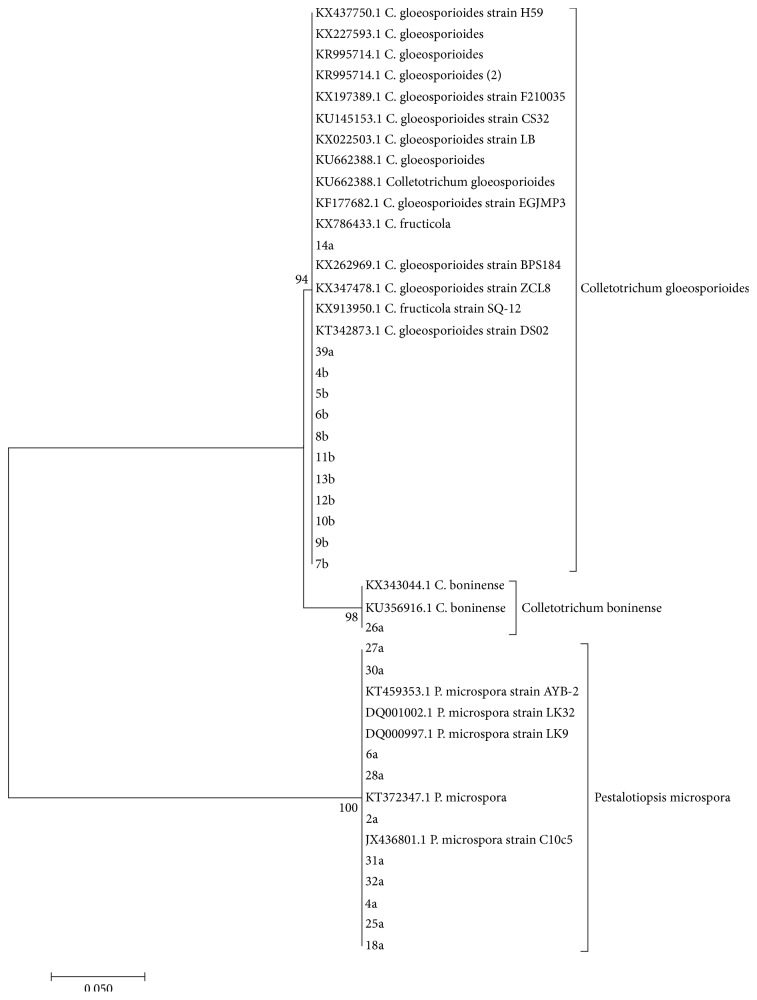
Maximum likelihood tree showing the relationship of* Colletotrichum gloeosporioides complex* and* Pestalotiopsis microspora* isolates based on ITS region.

The molecular identification of* C. gloeosporioides* and* Pestalotiopsis* spp. was inferred from 13 sequences of* C. gloeosporioides* and 10 sequences of* Pestalotiopsis* isolates. A phylogenetic tree (Figure 1) was made of sequences from* C. gloeosporioides, Pestalotiopsis microspora*, and reference sequences from the Genbank (Figure 3). The identity of the isolates of both* Colletotrichum* and* Pestalotiopsis* to the Genbank isolates ranged from 98% to 100%.

The phylogenetic analysis of the isolates and references from the Genbank resulted into three clades: Clade 1* (Colletotrichum gloeosporioides)*, Clade 2* (Colletotrichum boninense)*, and Clade 3* (Pestalotiopsis microspora)* (Table 4 and Figure 3).

## 4. Discussion and Conclusion

### 4.1. Cultural and Morphological Characteristics of* Colletotrichum gloeosporioides* and* Pestalotiopsis microspora* Isolates

The fungal isolates from diseased avocado fruits showing symptoms of anthracnose collected from the study area varied significantly in their cultural characteristics on PDA media in terms of texture and colour. A total of 46 isolates had whitish, greyish, or creamish colour and cottony, velvety mycelium on the top side and greyish cream with circular orange-pinkish colour on the reverse side. Similar cultural characteristics among* Colletotrichum gloeosporioides* isolates from avocado fruits were observed by [31]. Further, Chowdappa et al. [32] also noted the wide cultural variations among* C. gloeosporioides* isolates. The mycelial growth, however, had uniform radial growth characterised by circular ring-like patterns common to* C. gloeosporioides.* Sharma and Kulshrestha [4] reported similar mycelial growth characteristic of* C. gloeosporioides *in vitro. The cultures produced spores which were straight with rounded end, ranging within 3.0–5.0 *μ*m in width and 10.3–18.2 *μ*m in length, characteristic of* Colletotrichum gloeosporioides* as also reported by Chowdappa et al. [32].

The remaining 34 isolates had whitish cream mycelium with black fruiting structure, acervuli on the upper side, and had light orange to orange at lower side. These isolates produced spores having 3-4 septa and 2 or 3 appendages, characteristics of* Pestalotiopsis microspora* as reported by El-argawy [33]. Further molecular characteristic of these isolates using universal primers ITS1 and ITS4 confirmed the species as* Pestalotiopsis microspora *as discussed below in molecular characterisation section.

The differences observed in cultural and morphological characters of the isolates could be associated with their genetic variations and repeated subculturing [34, 35]. Further, the isolates had significantly different growth rate at *P* < 0.05 (Table 2). Such growth rate among* C. gloeosporioides* isolates was also reported by Zakaria and Bailey [31]. Overall the proportion in percentage (%) of the variance in mycelia radial diameter that was predictable from days for the isolates varied from the lowest (*R*2 = 0.812) to highest value (*R*2 = 0.993).

In this study, sporulation by* Colletotrichum gloeosporioides* isolates exhibited a wide range of mean number of spores per isolate from lowest 0.67 × 10^6^ to the highest 9.00 × 10^6^ spores per ml (Table 3) similar to observation made by Peres et al. [9]. These mean numbers of spores per ml differed significantly at *P* < 0.05 among isolates (Table 3).

The spores observed in this study were cylindrical and straight with smooth round end. Similar spores of* C. gloeosporioides* were observed by Chowdappa et al. [32]. The spore size varied significantly at *P* < 0.05 among isolates ranging within 3.0–5.0 *μ*m in width and 10.3–18.2 *μ*m in length (Table 3). Overall, spore size in terms of width and length differed significantly at *P* < 0.05 among isolates.

### 4.2. Phylogenetic Studies of* Colletotrichum gloeosporioides* and* Pestalotiopsis microspora*

Phylogenetic results showed that ribosomal internal transcribed spacers (ITS) DNA can be used to indicate the relationships within* Colletotrichum* and* Pestalotiopsis* species. The study identified* Colletotrichum gloeosporioides* and* Colletotrichum boninense* based on a randomly selected sample of 13 sequences of* Colletotrichum gloeosporioides* isolates using ITS4 (universal) and CgInt (*C. gloeosporioides* specific) primers yielding single band of 450 bp and 98–100% homology with nucleotide sequence of ITS region of DNA with* C. gloeosporioides* isolates in the Genbank (Figure 1). This was in agreement with the nucleotide sequence of ITS region of ribosomal DNA of* Colletotrichum gloeosporioides* isolates from orchids amplified using specific primers CgInt and ITS4 as reported by Chowdappa et al. [32]. Further, molecular identification of* Pestalotiopsis microspora* was based on 10 randomly selected sequences of* Pestalotiopsis microspora* isolates using universal ITS1 and ITS4 primers for DNA sequencing.

Twelve isolates of* C. gloeosporioides*, one isolate of* C. boninense*, and ten isolates of* P. microspora* from the study area gave identical sequences of the published sequences in the Genbank for the same species with 94%, 98%, and 100% bootstraps value, respectively (Figure 1).* Colletotrichum gloeosporioides* identified in this study has been reported as the most common and wide spread pathogen in all avocado growing region worldwide [36–38]. Further it has been associated with infection of other hosts such as almond, coffee, guava, apple, dragon fruit, cassava, mango, sorghum, and strawberry [2, 15, 39]. Though* C. boninense *identified is not very common in the study area, its sequence was identical to published sequence (KX 343044.1 and KU356916.1) in the Genbank. This species, among others like* Colletotrichum acutatum*,* Colletotrichum godetiae*,* C. fioriniae, C. aenigma, *and* Colletotrichum gigasporum, *has been reported to cause anthracnose of avocado [11, 16, 40, 41].

The ten randomly sampled isolates of* P. microspora *identified in this study showed 100% identity with the published sequences in the Genbank thereby confirming their identity (Figure 1).* Pestalotiopsis* species are widespread in both tropical and temperate region [42, 43]. Individual species of* Pestalotiopsis* are known to cause infection on a wide range of hosts [44, 45].* Pestalotiopsis clavispora* is known to cause stem end rot of avocado [6];* P. versicolor* has been reported as a causal agent of anthracnose in avocado [46].* P. palmarum* known to cause leaf spot and fruit canker in avocado [47]. Though* P. microspora* is prevalent in both tropics and subtropics, its association with host plants is not well researched [48]. It has been regarded both as an endophyte and as a pathogen causing postharvest diseases [49]. It has been reported to cause scab disease of guava fruits in Hawaii [48]. In this study, the fungus was isolated from diseased avocado fruits showing symptoms associated with anthracnose disease of avocado. However, despite its prevalence,* P*.* microspora *and its role in plant ecology are poorly understood [49].

Conclusively,* Colletotrichum gloeosporioides, Colletotrichum boninense, *and* Pestalotiopsis microspora* were identified for the first time as the causal agents of anthracnose of avocado in Kenya through cultural, morphological, and molecular techniques.

## Figures and Tables

**Table 1:** Cultural and morphological characteristics of the *Colletotrichum  gloeosporioides* on PDA.

Number of isolates	Upper side		Lower side	Zonation	Conidial shape
	Colony colour	Texture	Colour		
24	Whitish grey	Cottony	Creamish grey	Concentric	Cylindrical and straight
12	Whitish cream	Velvety	Greyish orange	Concentric	Cylindrical and straight
10	Greyish pink	Velvety	Grey	Concentric	Cylindrical and straight

**Table 2:** Daily mycelial growth and mean number of spores of *Colletotrichum  gloeosporioides* isolates.

Isolate	Day 2	Day 4	Day 6	Day 8	Day 10	Mean number of spores (10^6^ ml)
s1	0.77^*∗*bcd^	1.10^jklmn^	1.33^pq^	2.57^l^	3.57^m^	9.00^*∗*a^
s40	0.63^defgh^	1.02^mno^	1.60^no^	3.05^ijk^	4.00^efghij^	6.00^b^
s44	0.57^fgh^	1.67^d^	2.23^def^	3.77^ab^	4.03^efghi^	5.67^bc^
s3	0.63^defgh^	1.02^mno^	1.60^no^	3.05^ijk^	4.00^efghij^	5.33^bc^
s5	0.57^fgh^	1.67^d^	2.23^def^	3.77^ab^	4.03^efghi^	5.00^bc^
s6	0.65^cdefgh^	1.43^efg^	2.12^efg^	3.45^bcdefgh^	4.23^bcde^	5.00^bcd^
s19	0.63^defgh^	1.47^ef^	2.33^de^	3.77^ab^	4.17^defg^	5.00^bcd^
s21	0.53^gh^	1.03^lmno^	1.73^lmn^	3.30^defghij^	3.90^hijkl^	5.00^bcd^
s10	0.50^h^	1.17^ijklm^	1.83^ijklm^	3.23^fghij^	3.80^ijklm^	5.00^bcd^
s18	0.63^defgh^	1.10^jklmn^	1.97^ghijk^	3.40^cdefgh^	4.07^efgh^	4.67^bcde^
s25	0.63^defgh^	1.10^jklmn^	1.97^ghijk^	3.40^cdefgh^	4.07^efgh^	4.67^bcde^
s28	0.60^efgh^	0.93^no^	1.20^q^	1.47^n^	2.60^n^	4.67^bcde^
s14	0.60^efgh^	0.93^no^	1.20^q^	1.47^n^	2.60^n^	4.33^bcde^
s17	0.60^efgh^	0.87^o^	1.73^lmn^	3.63^abcd^	4.17^defg^	4.33^bcde^
s2	0.60^efgh^	0.87^o^	1.73^lmn^	3.63^abcd^	4.17^defg^	4.00^cdef^
s22	0.50^h^	1.43^efg^	2.03^fghi^	3.67^abc^	4.07^efgh^	4.00^cdef^
s24	0.70^cdef^	1.27^ghij^	1.97^ghijk^	3.63^abcd^	4.10^efgh^	4.00^cdef^
s29	0.60^efgh^	1.20^hijkl^	1.70^lmno^	3.70^abc^	4.00^efghij^	3.67^def^
s30	0.73^bcde^	1.37^fgh^	2.03^fghi^	3.60^abcde^	4.20^cdef^	3.33^defg^
s43	0.87^ab^	1.57^de^	2.80^b^	3.70^abc^	4.03^efghi^	3.33^defg^
s13	0.60^efgh^	1.10^jklmn^	1.65^mno^	2.82^kl^	4.37^abcd^	3.33^defg^
s15	0.60^efgh^	1.00^mno^	1.60^no^	2.97^jk^	4.03^efghi^	3.17^efg^
s4	0.67^cdefg^	1.20^hijkl^	1.97^ghijk^	3.43^bcdefgh^	4.13^Defgh^	3.00^efg^
s8	0.93^a^	1.90^c^	3.23^a^	3.57^bcdef^	4.10^Efgh^	3.00^efg^
s38	0.50^h^	1.43^efg^	2.03^fghi^	3.30^defghij^	4.50^a^	3.00^efg^
s16	0.63^defgh^	2.18^b^	2.37^cd^	3.28^efghij^	3.65^Lm^	3.00^efg^
s26	0.80^abc^	1.13^jklm^	1.88^hijkl^	3.18^ghij^	3.90^Hijkl^	3.00^efg^
s45	0.80^abc^	1.90^c^	2.58^bc^	3.15^hijk^	3.93^Ghijk^	3.00^efg^
s9	0.67^cdefg^	1.32^fghi^	2.08^fgh^	3.43^bcdefgh^	4.02^Efghij^	2.67^fg^
s12	0.70^cdef^	1.37^fgh^	2.10^fgh^	3.37^cdefghi^	4.43^Abc^	2.67^fg^
s20	0.77^bcd^	1.23^hijk^	1.60^no^	3.13^hijk^	3.97^Fghij^	2.33^fgh^
s27	0.80^abc^	2.63^a^	3.40^a^	3.93^a^	4.13^Defgh^	2.33^fgh^
s31	0.60^efgh^	1.03^lmno^	1.50^op^	2.50^l^	3.77^Jklm^	2.33^fgh^
s32	0.57^fgh^	0.93^no^	1.33^pq^	2.03^m^	2.37^n^	2.33^fgh^
s33	0.30^i^	0.40^p^	0.97^r^	1.43^n^	2.60^n^	2.33^fgh^
s35	0.70^cdef^	1.03^lmno^	1.70^lmno^	3.25^fghij^	4.07^Efgh^	2.00^gh^
s36	0.70^cdef^	1.17^ijklm^	1.97^ghijk^	3.63^abcd^	4.10^Efgh^	2.00^gh^
s41	0.63^defgh^	1.10^jklmn^	1.77^klmn^	2.83^kl^	4.00^Efghij^	2.00^gh^
s7	0.60^efgh^	1.13^jklm^	1.33^pq^	2.53^l^	3.80^Ijklm^	1.00^h^
s11	0.57^fgh^	1.07^klmn^	2.00^ghij^	3.50^bcdefg^	4.47^Ab^	1.00^h^
s23	0.70^cdef^	1.13^jklm^	1.77^klmn^	3.50^bcdefg^	4.47^Ab^	0.67^h^
s34	0.77^bcd^	1.07^klmn^	1.78^jklmn^	3.27^efghij^	4.03^Efghi^	0.67^h^
s39	0.77^bcd^	1.07^klmn^	1.67^lmno^	1.87^m^	4.03^Efghi^	0.66^h^
s42	0.77^bcd^	1.07^klmn^	1.67^lmno^	1.87^m^	4.03^Efghi^	0.66^h^
s37	0.77^b–d^	1.13^jklm^	1.63^mno^	2.97^jk^	3.70^Klm^	0.65^hi^
s46	0.70^c–f^	1.58^de^	2.67^b^	3.55^bcdef^	4.22^Bcdef^	0.65^hi^
LSD (0.05)	0.16	0.17	0.23	0.34	0.25	1.673
*P*-value	<0.001	<0.001	<0.001	<0.001	<0.001	<0.001

^*∗*^Data are means of three replicates. Means on the same column followed by similar letter(s) are not significantly different at *P* ≥ 0.05 according to Fisher's protected LSD test.

**Table 3:** The mean width and length of spores in micron (*μ*m) produced by 10-day-old *Colletotrichum  gloeosporioides* isolates.

Isolate	Width	Length
s1	5.0^*∗*a^	18.2^a^
s40	5.0^a^	18.0^a^
s44	5.0^a^	18.0^a^
s3	4.7^a^	17.1^ab^
s5	4.7^a^	17.1^ab^
s6	4.7^a^	16.2^bc^
s19	3.7^b^	16.1^bc^
s21	3.7^b^	16.1^bc^
s10	3.6^bc^	16.0^bc^
s18	3.6^bc^	15.4^cd^
s25	3.6^bc^	15.3^cd^
s28	3.6^bc^	15.2^cde^
s14	3.5^bcd^	15.2^cde^
s17	3.5^bcd^	15.2^cde^
s2	3.5^bcd^	15.1^cde^
s22	3.5^bcd^	15.0^cde^
s24	3.5^bcd^	15.0^cde^
s29	3.5^bcd^	15.0^cde^
s30	3.5^bcd^	15.0^cde^
s43	3.5^bcd^	14.5^def^
s13	3.4^bcde^	14.4^defg^
s15	3.4^bcde^	14.2^defgh^
s4	3.4^bcde^	14.0^efgh^
s8	3.4^bcde^	14.0^efgh^
s38	3.3^bcde^	13.5^fgh^
s16	3.3^bcde^	13.2^ghi^
s26	3.3^bcde^	13.2^ghi^
s45	3.3^bcde^	13.2^ghi^
s9	3.3^bcde^	13.1^hi^
s12	3.2^cde^	13.1^hi^
s20	3.2^cde^	13.0^hi^
s27	3.2^cde^	12.2^ij^
s31	3.2^cde^	12.2^ij^
s32	3.2^cde^	12.2^ij^
s33	3.2^cde^	12.1^ij^
s35	3.2^cde^	12.1^ij^
s36	3.2^cde^	12.1^ij^
s41	3.2^cde^	12.1^ij^
s7	3.2^cde^	12.0^ij^
s11	3.1^de^	12.0^ij^
s23	3.1^de^	11.1^jk^
s34	3.1^de^	11.0^jk^
s39	3.1^de^	11.0^jk^
s42	3.1^de^	10.4^k^
s37	3.0^e^	10.3^k^
s46	3.0^e^	10.3^k^
LSD	0.4031	1.2096
*P* value	<0.001	<0.001

^*∗*^Data are means of three replicates. Means on the same column followed by similar letter(s) are not significantly different at *P* ≥ 0.05 according to Fisher's protected LSD test.

**Table 4:** * Colletotrichum* and *Pestalotiopsis* isolates with their Genbank accession number.

Species	Culture	Location	Genbank number
*Colletotrichum gloeosporioides*	14^a^	Murang'a County	MG013524
	39^a^		MG013525
	4^b^		MG013527
	5^b^		MG013528
	6^b^		MG013529
	8^b^		MG013530
	10^b^		MG013531
	11^b^		MG013532
	12^b^		MG013533
	13^b^		MG013534
	9^b^		MG013535
	7^b^		MG013536

*Colletotrichum boninense*	26^a^	Murang'a County	MG013526

*Pestalotiopsis microspora*	27^a^	Murang'a County	MG013537
	30^a^		MG013538
	6^a^		MG013539
	28^a^		MG013540
	2^a^		MG013541
	31^a^		MG013542
	32^a^		MG013543
	4^a^		MG013544
	25^a^		MG013545
	18^a^		MG013546
